# Secondary Metabolites and Biological Activities of *Talaromyces sp. *LGT-2*, *an Endophytic Fungus from *Tripterygium Wilfordii*

**Published:** 2016

**Authors:** Qiu-Hong Zhao, Zhong-Duo Yang, Zong-Mei Shu, Yong-Gang Wang, Ming-Gang Wang

**Affiliations:** *School of Life Science a Engineering, Lanzhou University of Technology, Lanzhou.*

**Keywords:** endophytic fungus, secondary metabolites, monoamine oxidase inhibition, *Talaromyces*, *Tripterygium wilfordi*

## Abstract

In the present study, eleven compounds (1-11) including nine alkaloids (1-9), one triterpenoid saponin (10) and one formamide (11) were isolated from *Talaromyces sp. *LGT-2, an endophytic fungus from *Tripterygium wilfordi*. Their structures were determined based on NMR and ESI-MS spectral data, as well as comparing previous literature data. This is the first report of the isolation of alkaloids (1-9) from *Talaromyces* genus. In the next step, all compounds were screened for their anti-monoamine oxidase, anti-acetylcholinesterase, antibacterial and antitumor activities. Compound 11 showed moderate anti-monoamine oxidase activity with IC_50_ value of 61 μM; compounds 3, 4, 8 showed weaker anti-acetylcholinesterase activity; compounds 1, 3, 4, 7, 8, 9 showed moderate antibacterial activities; compounds 7, 8, 9 showed cytotoxicity against B16 cancer cell line with inhibitory rate of 86%, 82%, 78%, respectively, at the concentration of 500 μg/mL.

## Introduction

Endophytic fungi have been proved to be a new source for natural compounds, literature reports that we can get secondary metabolites which have unique structure and wide range of biological activities, such as antitumor, antimicrobial and antituberculosis. Indeed , structural diversity of these metabolites make endophytic fungi a potential new lead for drug discovery and development (1, 2).

**Figure 1:**
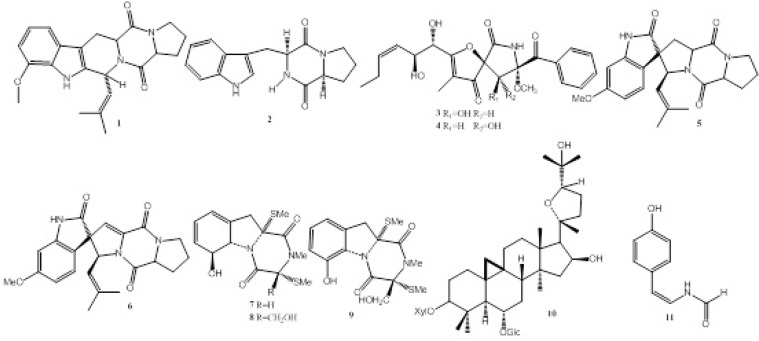
The structures of compounds 1-11

During our ongoing screening for new bioactive natural products from endophytes, we found the fermentation broth of *Talaromyces sp. *LGT-2 (GenBank Accession No. KF934203), an endophytic fungus inhabited in *Tripterygium wilfordi*, showed moderate monoamine oxidase (MAO) inhibitory activity with IC_50_ value of 85 μg/mL. Further chemical investigation resulted in the isolation of compounds 1-11 (Figure 1.). Anti-MAO activity, anti-acetylcholinesterase (anti-AChE), antitumor and antibacterial activities of compounds 1-11 were also evaluated in this study (Table 1.).

## Experimental

Chemicals and Instrumentation**: **Column Chromatography (CC): was performed on silica gel (200–300 mesh) and Sephadex LH-20 gel. HPLC was performed on JASCO liquid chromatograph with C_18_ column. TLC: was carried out on silica gel GF254 by using various solvent systems. The structures of the compounds were determined based on their NMR and ESI-MS spectroscopy.

Fungus Material: Chinese medicine* Tripterygium wilfordii* was purchased from the local market in GanSu province of China in December 2013, and authenticated by Professor LinYang (School of Life Science and Engineering, Lan Zhou University Of Technology, Lan Zhou, China). 

The strain LGT-2 was isolated from Chinese herb medicine *Tripterygium wilfordii *and was identified as *Talaromyces sp.* based on both morphology on PDA and analysis of the DNA sequences of the ITS1-5.8S-ITS2 ribosomal DNA gene region (GenBank Accession No. KF934203). 

Extraction and Isolation: The fungus LGT-2 was cultured in potato-dextrose broth (PDB) for 20d at 28 ^0^C on a 50 L fermenter. The fermentation broth was filtered .0 mg). Fr. 5 was purified by semipreparative HPLC (MeOH–H_2_O, 40:60) to yield compound 5 (5.6 mg), 6 (6.5 mg), 9 (8.5 mg).

Antimicrobial assay: The antimicrobial assay was performed by measuring zones of inhibition (mm) using standard disc diffusion technique (3). A positive control, amoxicillin (0.1mg/mL) was used for comparison purpose, whilst a blank disc impregnated with appropriate solvent was used as a negative control. In addition, the minimum inhibitory concentrations (MIC) of all the monomer compounds against* Escherichia coli, Pseudomonas Aeruginosa, Staphylococcus aureus, Bnfillus licheniformis, Streptococcus pneumoniae*, were determined by serial dilution technique.

Cytotoxicity Bioassay: The cytotoxicity of compounds 1-11 against B16 cancer cell line was measured by the MTT method(4). Cyclophosphamide was used as positive control. 

Anti-MAO and anti-AChE Bioassay: The procedure of testing MAO and AChE inhibiting activity was same with that reported in our previous paper (5, 6). 

**Table 1:** Anti-bacterial activity of monomer compounds (MIC, mg/mL).

Sample	*Escherichia coli*	*Pseudomonas Aeruginosa*	*Staphylococcus aureus*	*Bnfillus licheniformis*	*Streptococcus pneumoniae*
**1**	0.5	0.8	0.25	0.25	0.125
**3**	0.5	0.5	0.5	0.125	1
**4**	0.5	0.5	0.5	0.25	0.125
**7**	0.25	0.25	0.125	0.125	0.125
**8**	0.5	0.8	0.25	0.25	0.125
**9**	0.5	0.5	0.5	0.25	1

## Results and Discussion

This study was focused on compounds isolated from second metabolites of *Talaromyces sp. *LGT-2, and evaluated biological activities. The methods of Column Chromatography and HPLC Chromatograph were simple and rapid for separation and purification of natural compounds. In the present study, eleven compounds (1-11) including nine alkaloids (1-9), one triterpenoid saponin (10) and one formamide (11) were isolated from *Talaromyces sp. *LGT-2. This is the first report of the isolation of alkaloids (1-9) from *Talaromyces* genus. Compound 11 showed moderate anti-monoamine oxidase activity with IC_50_ value of 61μM, therefore, it was proved to be the responsible compound of anti-MAO activity; compounds 3, 4, 8 showed weaker anti-acetylcholinesterase activity; compounds 1, 3, 4, 7, 8, 9 showed moderate antibacterial activity (Table 1.); compounds 7, 8, 9 showed weak cytotoxicity against B16 cancer cell line with inhibitory rate of 86%, 82%, 78%, respectively, at the concentration of 500 μg/mL.

**Structure elucidation of the isolated compounds:**

Fumitremorgin C (1). Colorless amorphous powder. EI-MS* m/z* (%): 379 (80) [M]^+^, 364 (14) [M-CH_3_]^+^, 324 (32), 281 (100), 212 (67).^1^H-NMR (400, CDCl_3_, δ, ppm, *J*/Hz ): 7.81 (1H, s, H-1), 7.43 (1H, d, *J *= 8.0, H-16), 6.86 (1H, s, H-19), 6.80 (1H, d, *J *= 8.0, H-17), 5.98 (1H, d, *J *= 9.2, H-3), 4.90 (1H, d, *J *= 9. 2, H-21), 4.18 (1H, dd,* J *= 11.6, 4.8, H-12), 4.11 (1H, t, *J *= 8.0, H-6), 3.83 (3H, s, OMe), 3.64 (2H, m, H-9), 3.50 (1H, dd,* J *= 16.0, 4.8, H-13a), 3.09 (1H, dd, *J *= 16.0, 11.6, H-13b), 2.38 (1H, m, H-7a), 2.25 (1H, m, H-7b), 2.06(1H, m, H-8a), 1.99 (3H, s, H-24), 1.89 (1H, m, H-8b), 1.68 (3H, s, H-23); ^13^C-NMR (100 MHz, CDCl_3_, δ, ppm ): 169.5 (C-5), 165.7 (C-11), 156.6 (C-18), 137.0 (C-20), 133.9 (C-22), 132.2 (C-2), 124.2 (C-21), 120.7 (C-15), 118.8 (C-16), 109.5 (C-17), 106.3 (C-14), 95.3 (C-19), 59.2 (C-6), 56.8 (C-12), 55.7 (OMe), 51.0 (C-3), 45.4 (C-9), 28.6 (C-7), 25.7 (C-23), 23.0 (C-8), 21.9 (C-13), 18.1 (C-24)^[7]^.

Brevianamide F (2). White amorphous powder. EI-MS* m/z* (%): 283 (10) [M]^+^, 185 (8), 130 (100), 84 (20), 43 (17). ^1^H-NMR (400 MHz, CDCl_3_, δ, ppm, *J*/Hz): 8.61 (1H, s, -NH), 7.58 (1H, d,* J *= 8.0, H-7), 7.38 (1H, d,* J *= 8.0, H-4), 7.24 (1H, t,* J *= 8.0, H-6), 7.14 (1H, t,* J *= 8.0, H-5), 7.05 (1H, s, H-2), 5.86 (1H, s, -NH), 4.36 (1H, d, *J *= 9.2, H-9), 4.06 (1H, t, *J *= 8.0, H-12), 3.76 (1H, dd, *J *= 1.2, 14.4, H-8b), 3.63 (2H, m, H-15), 2.97 (1H, dd, *J *= 9.2, 14.4, H-8a), 1.86-2.34 (4H, m, H-16, 17); ^13^C-NMR (100 MHz, CDCl_3_, δ, ppm): 169.4 (C-11), 165.5 (C-14), 136.6 (C-7a), 126.6 (C-3a), 123.4 (C-2), 122.7 (C-5), 119.7 (C-6), 118.5 (C-4), 111.5 (C-7), 109.5 (C-3), 59.1 (C-12), 54.5 (C-9), 45.3 (C-15), 28.2 (C-17), 26.8 (C-8), 22.5 (C-16)(8).

Pseurotin A_1_ (3). Light yellow oil. [α]_D_^25 ^–4.8 (c 0.1, MeOH). ESI-MS *m/z* 454.1 [M + Na]^+^. ^1^H-NMR (400 MHz, DMSO-d_6_, δ, ppm, *J*/Hz): 9.94 (1H, s, NH), 8.10 (2H, d,* J *= 8.0 Hz, H-19, 23), 7.63 (1H, t,* J *= 7.3, H-21), 7.52 (2H, t,* J *= 8.0, H-20, 22), 6.07 (1H, d,* J *= 6.2, 9-OH), 5.75 (1H, d,* J *= 5.5, 10-OH), 5.42 (1H, dd,* J *= 7.0, 11.0, H-13), 5.39 (1H, dd,* J *= 8.1,11.0, H-12), 4.96 (1H, d,* J *= 5.1, 11-OH), 4.62 (1H, d,* J *= 6.2, H-9), 4.45 (1H, dd, *J *= 5.2, 11.0, H-11), 4.36 (1H, t,* J *= 5.2, H-10), 3.12 (3H, s, OMe-8), 2.01 (1H, m, H-14), 1.95(1H, m, H-14), 1.66 (3H, s, H-16), 0.86 (3H, t,* J *= 7.2, H-15); ^13^C-NMR (100 MHz, DMSO-d_6_, δ, ppm): 201.4 (C-4), 194.6 (C-17), 187.2 (C-2), 168.1 (C-6), 135.8 (C-13), 134.6 (C-18), 133.9 (C-21), 130.5 (C-19), 130.5 (C-23), 129.4 (C-12), 128.1 (C-20), 128.1 (C-22), 113.1 (C-3), 97.6 (C-8), 89.5 (C-5), 77.0 (C-9), 72.7 (C-10), 69.9 (C-11), 52.1 (OMe-8), 22.3 (C-14), 14.4 (C-15), 5.8 (C-16)(9).

Pseurotin A_2_ (4). Light yellow oil. [α]_D_^25 ^–30.0 (c 0.1, MeOH). ESI-MS *m/z* 454.1 [M + Na]^+^. ^1^H-NMR (400 MHz, DMSO-d_6_, δ, ppm, *J*/Hz): 9.98 (1H, s, NH), 8.28 (2H, d,* J *= 8.0, H-19, 23), 7.69 (1H, t,* J *= 7.3, H-21), 7.55 (2H, t,* J *= 8.0, H-20, 22), 6.36 (1H, d,* J *= 10.0, 9-OH), 5.88 (1H, d,* J *= 5.5, 10-OH), 5.47 (1H, dt,* J *= 7.0, 11.0, H-13), 5.41 (1H, dd,* J *= 8.1, 11.0, H-12), 5.10 (1H, d,* J *= 5.1, 11-OH), 4.18 (1H, d,* J *= 10.0, H-9), 4.57 (1H, dd, *J *= 5.2, 8.1, H-11), 4.51 (1H, t,* J *= 5.2, H-10), 3.21 (3H, s, OMe-8), 2.07 (2H, m, H-14), 1.66 (3H, s, H-16), 0.93 (3H, t*, J *= 7.2, H-15); ^13^C-NMR (100 MHz, DMSO-d_6_, δ, ppm): 199.8 (C-4), 196.1 (C-17), 188.8 (C-2), 169.2 (C-6), 135.7 (C-13), 134.6 (C-18), 133.9 (C-21), 130.5 (C-19), 130.5 (C-23), 129.4 (C-12), 128.1 (C-20), 128.1 (C-22), 114.0 (C-3), 95.4 (C-8), 88.0 (C-5), 75.8 (C-9), 71.9 (C-10), 69.6 (C-11), 51.7 (OMe-8), 21.7 (C-14), 14.4 (C-15), 5.8 (C-16)^[9]^. 

Spirotryprostatin A (5). Colorless acicular crystals. ESI-MS *m/z* 396.0 [M+H]^+^. ^1^H-NMR (400 MHz, CDCl_3_, δ, ppm, *J*/Hz): 7.51 (1H, s, H-1), 6.93 (1H, d, *J *= 8.4, H-4), 6.50 (1H, d, *J *= 8.4, H-5), 6.43 (1H, s, H-7), 5.00 (2H, m, H-18, 9), 4.77 (1H, *J *= 9.0, H-19), 4.29 (1H, t, *J *= 8.4, H-12), 3.80 (3H, s, -OMe), 3.68 (2H, m, H-15), 2.60 (1H, dd, *J *= 10.8, 13.2, H-13b), 1.95-2.41 (7H, m, H-13a, 14, 15, 8), 1.59 (3H, s, H-21), 1.25 (3H, s, H-22)(10).

6-Methoxyspirotryprostatin B (6). Colorless acicular crystals. ESI-MS *m/z* 392.2 [M-H]^-^. ^1^H-NMR (400 MHz, CDCl_3_, δ, ppm, *J*/Hz): 7.64 (1H, s, H-1), 6.95 (1H, d, *J *= 8.4, H-4), 6.51 (1H, d, *J *= 8.4, H-5), 6.44 (1H, s, H-7), 5.76 (1H, s, H-8), 5.38 (1H, d, *J *= 8.8, H-18), 5.19 (1H, d, *J *= 8.8, H-19), 4.34 (1H, dd, *J *= 10.0, 6.0, H-12), 3.80 (3H, s, -OMe), 3.83 (1H, m, H-15b), 3.55 (1H, m, H-15a), 2.48 (1H, m, H-13b), 2.12 (1H, m, H-14b), 1.98 (2H, m, H-13a, 14a), 1.59 (3H, s, H-21), 1.25 (3H, s, H-22)(11).

3-Dehydroxymethylbisdethio-3, 10a-bis(methylthio)gliotoxin(7).Colorless acicular crystals. ESI-MS *m/z* 349.0 [M + Na]^+^. ^1^H-NMR (400 MHz, CD_3_COCD_3_, δ, ppm, *J*/Hz): 2.17 (3H, s, -SMe), 2.43 (3H, s, -SMe), 2.86 (2H, brs, H-10), 3.11 (3H, s, -NMe), 4.71 (1H, d, *J *= 13.2, H-5a), 4.81 (1H, m, H-6), 5.63 (1H, m, H-7), 5.89 (1H, m, H-8), 5.99 (1H, brs, H-9); ^13^C-NMR (100 MHz, CD_3_COCD_3_, δ, ppm): 168.7 (C-1), 165.0 (C-4), 133.9 (C-9a), 131.2 (C-8), 123.8 (C-7), 120.2 (C-9), 75.0 (C-6), (C-9a) 72.8 (C-11), 70.0 (C-5a), 68.1 (C-3), 38.8 (C-10), 31.9 (-NMe), 17.7 (-SMe), 14.6 (-SMe)(12).

 Bisdethiobis(methylthio)gliotoxin (8). Light yellow oil. EI-MS* m/z* (%): 356 (10) [M]^+^, 309 (50) [M-SMe]+, 261 (100), 231 (75). ^1^H-NMR (400 MHz, CD_3_OD, δ, ppm, *J*/Hz): 2.24 (3H, s, -SMe), 2.27(3H, s, -SMe), 2.93 (1H, d,* J *= 14.6, H-10a), 3.12 (1H, d,* J *= 14.6, H-10b), 3.11 (3H, s, -NMe), 3.86 (1H, d,* J *= 11.6, H-15a), 4.24 (1H, d,* J *= 11.6, H-15b), 4.86 (1H, m, H-5a), 4.95 (1H, m, H-6), 5.67(1H, m, H-7), 5.92(2H, m, H-8, 9); ^13^C-NMR (100 MHz, CD_3_OD, δ, ppm): 168.3 (C-1), 167.7 (C-4), 134.0 (C-9a), 130.7 (C-8), 124.8 (C-7), 120.8 (C-9), 75.7 (C-6), 74.3 (C-3), 73.1 (C-11), 70.4 (C-5a), 64.6 (C-15), 39.7 (C-10), 29.1 (-NMe), 15.2 (-SMe), 13.6 (-SMe) (13). Didehydrobisdethiobis(methylthio)gliotoxin (9). Light yellow oil. EI-MS* m/z* (%): 354 (9) [M]^+^, 307 (79) [M-SCH_3_]^+^, 259 (100), 243 (41), 229 (88), 160 (58).^ 1^H-NMR (400 MHz, CDCl_3_, δ, ppm, *J*/Hz): 10.20 (1H, s, -OH), 7.16 (1H, t, *J *= 8.0, H-8), 6.89 (1H, d, *J *= 8.0, H-9), 6.81 (1H, d, *J *= 8.0,H-7), 4.52 (1H, d, *J *= 12.0, H-15b), 3.98 (1H, d, *J *= 12.0, H-15a), 3.60 (1H, d, *J *= 12.6, H-10b), 3.46 (1H, d, *J *= 12.6, H-10a), 3.21 (3H, s, -NMe), 2.33 (3H, s, -SMe), 2.25 (3H, s, -SMe)(14).

Cyclosieversioside F (10). Colorless amorphous powder. ^1^H-NMR (400 MHz, CD_3_OD, δ, ppm, *J*/Hz): 4.90 (1H, d, *J *= 7.6, H-1 of D-glucose), 4.65 (1H, d, *J *= 7.6, H-1 of D-xylose), 4.29 (1H, m, H-16), 3.13-3.85 (11H, m, D-xylose + D-glucose), 1.00, 1.01, 1.12, 1.20, 1.25, 1.25, 1.27 (each 3H, s, Me-18, 21, 26, 27, 28, 29, 30), 0.27 and 0.58 (each 1H, d, *J *= 4.0, H-19); ^13^C-NMR (100 MHz, CD_3_OD, δ, ppm): 107.4 (C-1’, Xyl), 104.9 (C-1’’, Glu), 90.0 (C-20), 88.4 (C-3), 82.5 (C-24), 80.0 (C-6), 78.6 (C-3’’, Glu), 77.7 (C-5’’, Glu), 75.6 (C-2’’, Glu), 75.5 (C-2’, Xyl), 74.7 (C-3’, Xyl), 74.7 (C-16), 71.8 (C-4’, Xyl), 71.3 (C-4’’, Glu), 71.3 (C-25), 66.7 (C-5’, Xyl), 62.9 (C-6’’, Glu), 58.9 (C-17), 53.3 (C-5), 46.7 (C-13), 47.0 (C-14), 46.7 (C-8), 46.1 (C-15), 43.1 (C-4), 35.4 (C-7), 35.1 (C-22), 34.2 (C-12), 33.0 (C-1), 30.4 (C-2), 29.6 (C-19), 28.2 (C-26), 29.6 (C-10), 28.5 (C-27), 27.0 (C-29), 27.6 (C-21), 26.5 (C-11), 26.8 (C-23), 22.1 (C-9), 21.5 (C-28), 20.2 (C-18), 16.6 (C-30)(15).

(Z)-N-(4-hydroxystyryl)formamide(11). Colorless acicular crystals. ESI-MS m/z 162.0[M-H]^+^. ^1^H-NMR ( CD_3_OD, 400 MHz, δ, ppm, *J*/Hz): 8.08 (1H, s, -CHO), 7.17 (2H, d, *J *= 8.0, H-2, H-6), 6.75 (1H, d, *J *= 9.6, H-7), 6.73 (2H, d, *J *= 8.0, H-3, H-5), 5.73 (1H, d, J = 9.6, H-8). ^13^C-NMR (100 MHz, CD_3_OD, δ, ppm): 118.0(C-1), 111.1(C-2), 126. 8 (C-1’), 130.2 (C-2’ /6’), 116.2(C-3’/5’), 157. 0 (C-4’), 160.7 (-CHO) (16). 
